# miR-2909-mediated regulation of KLF4: a novel molecular mechanism for differentiating between B-cell and T-cell pediatric acute lymphoblastic leukemias

**DOI:** 10.1186/1476-4598-13-175

**Published:** 2014-07-18

**Authors:** Deepti Malik, Deepak Kaul, Nalini Chauhan, Ram Kumar Marwaha

**Affiliations:** 1 Department of Experimental Medicine & Biotechnology, Postgraduate Institute of Medical Education & Research, Chandigarh, India; 2 Department of Advanced Pediatrics Centre, Postgraduate Institute of Medical Education & Research, Chandigarh, India; 3 Department of Experimental Medicine & Biotechnology, Research Block B, Postgraduate Institute of Medical Education & Research, Chandigarh 160012, India

**Keywords:** Acute lymphoblastic leukemia, miR-2909, Kruppel-like factor 4, Homology modeling, Cell cycle

## Abstract

**Background:**

microRNAs (miRNAs) play both oncogenic and oncostatic roles in leukemia. However, the molecular details underlying miRNA-mediated regulation of their target genes in pediatric B- and T-cell acute lymphoblastic leukemias (ALLs) remain unclear. The present study investigated the relationship between miR-2909 and Kruppel-like factor 4 (*KLF4*), and its functional relevance to cell cycle progression and immortalization in patients with pediatric ALL.

**Methods:**

Elevated levels of miR-2909 targeted the tumor suppressor gene *KLF4* in pediatric B-cell, but not pediatric T-cell ALL, as detected by pMIR-GFP reporter assay. Expression levels of genes including apoptosis-antagonizing transcription factor (*AATF*), *MYC*, B-cell lymphoma (*BCL3*), *P21*^*CIP*^, *CCND1* and *SP1* in B- and T-cells from patients with pediatric ALL were compared with control levels using real-time quantitative reverse transcription polymerase chain reaction, western blotting, and reporter assays.

**Results:**

We identified two novel mutations in *KLF4* in pediatric T-ALL. A mutation in the 3′ untranslated region of the *KLF4* gene resulted in loss of miR-2909-mediated regulation, while mutation in its first or third zinc-finger motif (Zf1/Zf3) rendered *KLF4* transcriptionally inactive. This mutation was a frameshift mutation resulting in alteration of the Zf3 motif sequence in the mutant *KLF4* protein in all pediatric T-ALL samples. Homology models, docking studies and promoter activity of its target gene *P21*^*CIP*^ confirmed the lack of function of the mutant *KLF4* protein in pediatric T-ALL. Moreover, the inability of miR-2909 to regulate *KLF4* and its downstream genes controlling cell cycle and apoptosis in T-cell but not in B-ALL was verified by antagomiR-2909 transfection. Comprehensive sequence analysis of *KLF4* identified the predominance of isoform 1 (~55 kDa) in most patients with pediatric B-ALL, while those with pediatric T-ALL expressed isoform 2 (~51 kDa).

**Conclusions:**

This study identified a novel miR-2909-*KLF4* molecular axis able to differentiate between the pathogeneses of pediatric B- and T-cell ALLs, and which may represent a new diagnostic/prognostic marker.

## Background

Acute lymphoblastic leukemia (ALL) is widely recognized as the most prevalent pediatric leukemia [1]; however, the genomic mechanisms responsible for the uncontrolled cell proliferation coupled with cell immortalization remain unknown [2]. In this context, the genes for apoptosis-antagonizing transcription factor (*AATF*) and Kruppel like factor 4 (*KLF4*) have assumed importance. *AATF* provides a critical link between cell cycle progression, check-point control, and apoptosis [3], and also encodes the novel microRNA (miRNA) miR-2909, which regulates genes involved in inflammation, cell cycle, and immune response [4-6]. *KLF4*, a member of the *SP1*/KLF transcription factor family, is characterized by three highly conserved C2H2-type zinc-finger motifs at its carboxyl terminus, which are crucial for its interaction with its target DNA [7]. The *KLF4* gene acts as both an oncogene and a tumor suppressor, depending on its genetic and cellular contexts [8]. The tumor-suppressive role of *KLF4* and its involvement in regulating apoptosis, proliferation, and differentiation in B-cell malignancies suggest that *KLF4* may play a critical role in leukemogenesis [9]. Furthermore, *KLF4* mRNA has been shown to be targeted by miR-130a and 135b in M1 acute myeloid leukemic blasts, and silencing of *KLF4* arrested the maturation of blood cells at an early progenitor stage [10].

The discovery of miRNAs has opened a new epigenomic dimension in terms of the understanding of oncogenesis in general and leukemogenesis in particular [11]. Alterations in miRNA expression patterns and their respective targets have been documented in various tumors [12] including different types of leukemias such as chronic lymphocytic leukemia [13], acute myeloid leukemia [14] and ALL [15], thus suggesting a possible correlation between miRNA expression status and the development of hematological malignancies.

The present study aimed to identify the expression status of *AATF*-encoded miR-2909 in B- and T-cells from patients with pediatric ALL and explore the possible relationship between miR-2909 and *KLF4* in these cells. We also investigated the functional importance of this relationship in the regulation of genes involved in cell cycle progression (*BCL3*, *CCND1*, MYC) and apoptosis (*AATF*). To the best of our knowledge, the results of this study provide the first evidence for miR-2909-dependent regulatory pathway as the possible underlying mechanism responsible for the initiation of ALL in humans.

## Results

### miR-2909 targets *KLF4*

**Figure 1:**
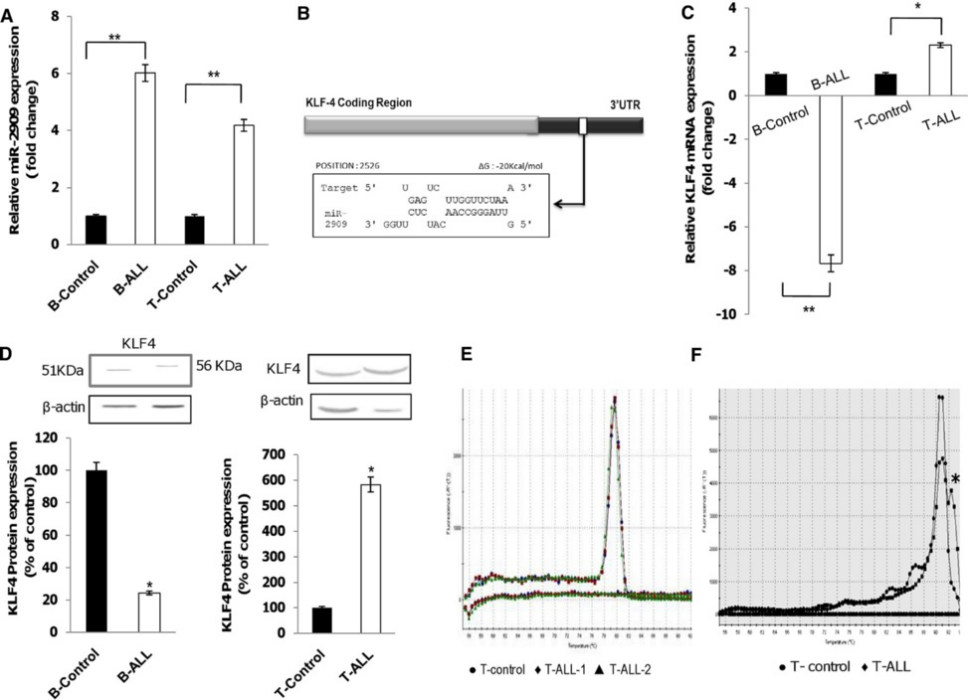
**miR-2909 and *KLF4 *expression in pediatric acute lymphoblastic leukemias*. *(A)** qRT-PCR analysis for relative expression of miR-2909 in patients with pediatric B-ALL and T-ALL compared with the corresponding controls. Expression was normalized to U6 snRNA; error bars represent mean ± S.D. n = 6, **P < 0.01 relative to B-and T-control. **(B)** Bioinformatics analysis of miR-2909 target site in the 3′UTR of *KLF4* gene **(C-D)***KLF4* expression level at the mRNA **(C)** and protein **(D)** in patients with pediatric ALL compared with the corresponding controls. β-actin was used as an invariant control; each bar represents mean ± S.D of the experiment performed in triplicate *P < 0.05 relative to B-and T-control; densitometric analysis of western blot results was performed using Scion Image Analysis Software. **(E-F)** Melting curve graphs of hsa-miR-2909 **(E)** and *KLF4* 3′UTR **(F)** respectively in pediatric T-ALL. Difference between melting curve graph for KLF4 3′UTR in T-ALL and control is indicated by asterisk (*).

Quantitative real-time polymerase chain reaction (PCR) analysis of miR-2909 expression in 30 pediatric patients with B-ALL and 20 with T-ALL revealed significantly increased expression levels compared with the corresponding controls (Figure 1A). To investigate the molecular mechanism of miR-2909 function in ALL, we performed *in silico* screening of genes reported to play crucial roles in leukemogenesis for the presence of miR-2909 target site(s) using an RNA hybrid tool (http://bibiserv.techfak.uni-bielefeld.de/rnahybrid/) [16]. Among all the identified genes, we focused on *KLF4* because it was reported to be significantly downregulated in ALL and functions as a tumor suppressor in B-cell hematological malignancies [9]. The 3′ untranslated region (UTR) region of *KLF4* harbored a target site for miR-2909 (Figure 1B). To validate this prediction experimentally, *KLF4* expression was observed at both the mRNA and protein levels in patients with ALL. Increased miR-2909 expression was always accompanied by significant downregulation of *KLF4* mRNA and protein in pediatric B-ALL compared with controls, indicating that miR-2909 may regulate the expression of *KLF4* by targeting its 3′UTR (Figure 1C and D). In contrast, both mRNA and protein expression levels of *KLF4* were upregulated in T-ALL compared with controls (Figure 1C and D), despite increased expression of miR-2909 in these T-ALL lymphoblasts (Figure 1A), suggesting the possibility of a mutation in either the seed sequence or the 3′UTR region of *KLF4* involving the miR-2909 binding site.

**Figure 2:**
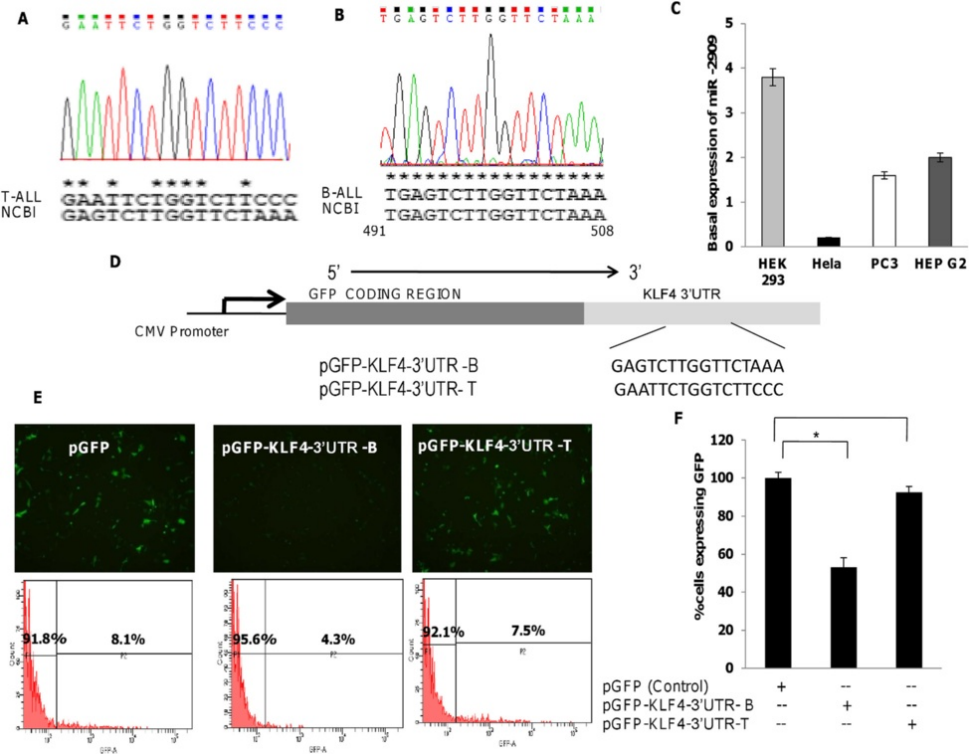
**miR-2909 targets 3′UTR of *KLF4 *in B-ALL but not T-ALL. (A-B)** The sequence alignment of the miR-2909 binding site, located at 491-508 nucleotides within *KLF4* 3′UTR region in pediatric T-ALL **(A)** and B-ALL **(B)**. The NCBI sequence is shown for comparison. **(C)** Endogenous expression of hsa-miR-2909 in indicated cancer cell lines representing high expression in HEK 293 cells and low expression in HeLa cells using qRT-PCR. **(D)** Schematics representing miRNASelect™ pMIR-GFP reporter vector construct containing KLF4 3′UTR and the miR-2909 target site from both B-ALL (pGFP-KLF4-3′UTR-B) and T-ALL (pGFP-KLF4-3′UTR-T) patients into the cloning site of the vector downstream of the green fluorescent protein (GFP) reporter gene under the control of CMV promoter. **(E)** Representative fluorescent microscopic images and FACS results of HEK 293 cells transfected with either control pGFP vector without 3′UTR insert or pGFP-*KLF4*-3′UTR-B reporter vector or pGFP-*KLF4*-3′UTR-T reporter vector following 48 h of transfection. **(F)** Percentage of cells expressing GFP in transfected cells with indicated constructs was calculated using flow cytometry. Each bar represents mean percentage ± S.D of cells expressing GFP of the experiment performed in triplicate; * P < 0.05 relative to control.

Melting-curve graphs for miR-2909 found no difference between T-ALL and controls, thus ruling out the presence of a mutation in the seed sequence of miR-2909 (Figure 1E). However, the melting-curve graph corresponding to the *KLF4* 3′UTR did show a significant difference (Figure 1F). In order to clarify these changes, amplicons corresponding to this region were sequenced and revealed the presence of a genetic aberration in the miR-2909-binding site of *KLF4* in T-ALL (Figure 2A). In contrast, no aberration was depicted in the miR-2909-binding site within the 3′UTR of *KLF4* in B-ALL (Figure 2B). To further confirm if miR-2909 targets *KLF4* in pediatric B-ALL but not in pediatric T-ALL, we constructed reporter plasmids using the *KLF4* 3′UTR containing the miR-2909 target site from B-ALL (pGFP-*KLF4*-3′UTR-B) and T-ALL (pGFP-*KLF4*-3′UTR-T) patients (Figure 2D). The 3′UTR regions of the *KLF4* genes from both B-ALL and T-ALL were amplified and inserted into the cloning site of the vector downstream of the green fluorescent protein (GFP) reporter gene. These plasmids were then transfected into HEK 293 cells, which intrinsically overexpressed miR-2909 compared with HeLa, PC3 and HepG2 cells (Figure 2C). HEK 293 cells carrying pGFP-*KLF4*-3′UTR-B exhibited a 52.07% reduction in GFP expression, whereas cells expressing pGFP-*KLF4*-3′UTR-T showed no noticeable difference compared with cells transfected with pGFP plasmid without 3′UTR inserts (Figure 2E and F). These results clearly revealed the ability of miR-2909 to repress *KLF4* expression in pediatric B-ALL, but not T-ALL.

### Sequence analysis of *KLF4*

**Figure 3:**
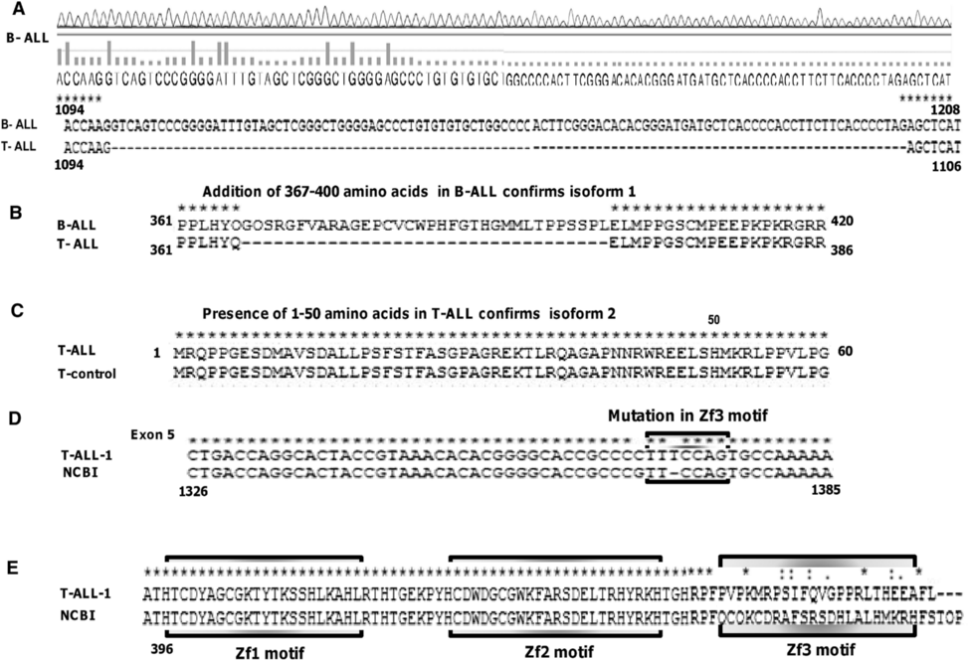
**Sequence analysis of *KLF4 *coding region in B-ALL and T-ALL samples. (A)** Snapshot of *KLF4* gene sequence depicting the predominance of a splice variant (isoform 1) of *KLF4* with the addition of 102 nucleotides between exons 3 and 4 in B-ALL samples. This entire region is absent in T-ALL samples. **(B)** Protein sequence depicting addition of 367-400 amino acids, confirming the presence of isoform 1 in B-ALL, while this entire region remains absent in all T-ALL samples in the present study **(C)** Presence of 1-50 amino acids confirms the existence of isoform 2 in all T-ALL study samples. The control sequence is shown for comparison. **(D)** Representative sequence data of pediatric T-ALL-1 sample is shown. A single nucleotide, T is inserted in the region corresponding to the third zinc-finger (Zf3) motif of KLF4 in T-ALL samples. **(E)** Genetic aberration(s) changed the entire reading frame, altering the sequence of KLF4 third zinc finger motif. The NCBI protein sequence is shown for comparison. Representative DNA and protein sequence alignment of KLF coding region (corresponding to three zinc finger motifs in exon 5) in other T-ALL samples are shown in Additional file 1: Figure S1.

UniProt gives three different isoforms of *KLF4*. Isoform 1 has the addition of 33 amino acids at location 367–400 while isoform 3 has a deletion of the first 50 amino acids (http://www.uniprot.org/uniprot/O43474) [17]. We aimed to determine if the isoforms of *KLF4* differed between B-ALL and T-ALL. The amplicons corresponding to full-length *KLF4* were sequenced in 30 B-ALL and 20 T-ALL samples. Strikingly, 25 B-ALL samples showed the presence of isoform 1, which incorporated an intronic region between exons 3 and 4 in the coding sequence resulting in an increased molecular mass of approximately 55 kDa (total length of 513 amino acids). The remaining five B-ALL and all 20 T-ALL samples showed the presence of isoform 2, containing 479 amino acids with a molecular mass of 51 kDa (Figure 3A and B).

We further explored if T-ALL patients expressed isoform 3 of *KLF-4*. Sequencing failed to identify any deletion of the first 50 amino acids in any of the T-ALL samples in the present study, confirming the absence of isoform 3 in these blast cells (Figure 3C). Interestingly, most T-ALL samples showed insertion/deletion of nucleotides in the region corresponding to the third zinc-finger (Zf3) motif of *KLF4*, while deletion of nucleotides in the first zinc-finger (Zf1) motif was identified in some T-ALL samples (Figure 3D, Additional file 1: Figure S1). These genetic aberrations changed the entire reading frame, altering the sequence of the *KLF4* zinc-finger motif and potentially destroying its DNA-binding affinity (Additional file 1: Figure S1G). Modeling/docking studies were performed using mutant *KLF4* lacking the Zf3 motif but with intact Zf1 and Zf2 motifs derived from pediatric T-ALL samples (Figure 3E). In addition, we also sequenced the *KLF4* coding region corresponding to the three zinc-finger motifs in B-ALL samples to detect the presence of any genetic aberration(s) in this region in pediatric B-ALL. Sequence analysis revealed no genetic aberrations in any of the three zinc-fingers regions of *KLF4* in samples from pediatric patients with B-ALL, suggesting that the conformation of *KLF4* was unaffected in these patients (Additional file 2: Figure S2).

### Molecular modeling and docking studies of *KLF4* protein

**Figure 4:**
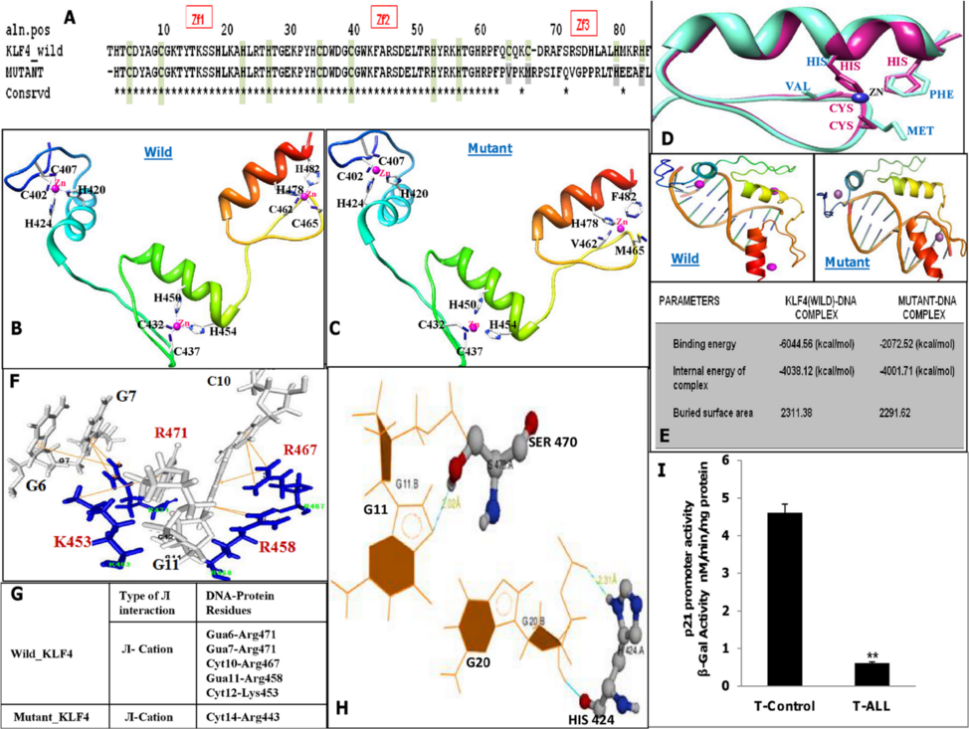
**Molecular Modeling and docking studies of wild-type and mutant *KLF4*. (A)** Representative sequence alignment of wild-type *KLF4* (derived from control T-cells) with mutant KLF4 (derived from pediatric T-ALL samples). The zinc finger motifs Zf1, Zf2 and Zf3 are highlighted and (*) indicates conserved residues. The grey region indicates third zinc finger motif (Zf3) with mutated amino acids. **(B-C)** The structure of zinc finger motifs for wild type **(B)** and mutant *KLF4***(C)** were built using MODELLER Program under Accelrys Discovery Studio version 2.5. Zf1; blue, Zf2; green and Zf3; red. Comparison of structural models of the third zinc-finger motif between wild-type and mutant KLF4 revealed the amino acid replacements C462V, C465M, and H482F, which were involved in coordination with zinc **(D)** Superimposition of Zf3 motif of wild-type (pink) and mutant *KLF4 (*blue*)*. **(E)** Molecular interaction of modeled wild-type and mutant KLF4 with its target DNA binding sequence with inserted table depicting the binding energies of the interacting complexes. **(F-G)** Docking analysis of wild-type KLF4 revealed that residues R458, K453, R471and R467 displayed cation-π interactions with guanine (at positions 11, 6, 7) and cytidine 10. In contrast, docking of mutant KLF4 with its target DNA sequence exhibited cation-π interactions only between R443 and cytidine 14. **(H)** Docking analysis of wild-type KLF4 revealed that residues H424 and S470 formed hydrogen bonds with guanine (at positions 20 and 11), respectively. Such interactions were missing in mutant *KLF4* as a consequence of altered sequence of KLF4 third zinc finger motif; R458 only formed a hydrogen bond with guanine 3. See Additional file 3: Figure S3 **(I)** β-galactosidase reporter activity in control and T-ALL lymphoblasts transfected with β-gal construct. The experiments were repeated thrice and results were reported as relative β-gal activity. *P < 0.05 relative to control.

Sequence alignment of wild-type *KLF4* (derived from control T cells) with mutant *KLF4* (derived from pediatric T-ALL samples) revealed that the Zf3 motif was mutated (Figure 4A). Comparison of structural models of the third zinc-finger motif between wild-type and mutant *KLF4* revealed the amino acid replacements C462V, C465M, and H482F, which were involved in coordination with zinc (Figure 4B, C and D). The structure of wild-type *KLF4* revealed the existence of a salt bridge between the guanidinium group of arginine residues (R449 and R471) and the surrounding anionic carboxylate (RCOO^-^) side chains of glutamic and aspartic acid (E446 and D473), respectively. The third glutamate residue (E446) formed a salt bridge with cationic ammonium (RNH_3_^+^) of lysine (E446-L453). In contrast, in mutant *KLF4*, the guanidinium (RNHC(NH_2_)_2_^+^) group of arginine only formed a salt bridge with the anionic carboxylate (RCOO^-^) of either glutamic acid or aspartic acid (E446 and R479, E446 and R479, D445–R443). The molecular interactions of modeled wild-type and mutant *KLF4* with a 10-bp DNA sequence (5′-cgggcggggc-3′) in the *P21*^*CIP*^ gene promoter, which is widely recognized as the transcriptional target site for *KLF4* protein [18,19], revealed that T-ALL mutant *KLF4* was unable to interact with its target DNA sequence as a result of mutation of the Zf3 motif, while wild-type *KLF4* exhibited proper cation-π and hydrogen-bonding interactions with its target DNA sequence (Figure 4E). Docking analysis of wild-type *KLF4* revealed that residues Arg458, Lys453, Arg471, and Arg467 displayed cation-π interactions with guanine (at positions 11, 6, 7) and cytidine 10 (Figure 4F and G), and residues His424 and Ser470 formed hydrogen bonds with guanine (at positions 20 and 11), respectively (Figure 4G and H). In contrast, docking of mutant *KLF4* with its target DNA sequence exhibited the formation of hydrogen bonds between Arg458 and guanine (at position 3) and cation-π interactions between Arg443 and cytidine14 (Figure 4G). The precise details of other non-covalent interactions are discussed in Additional file 3: Figure S3.

### Loss of transcriptional activity of mutant *KLF4* protein

To confirm the bioinformatics result suggesting that mutant *KLF4* was unable to interact with its 10-bp target sequence in the *P21*^*CIP*^ gene promoter in pediatric T-ALL, we transfected a β-galactosidase (β-gal) construct under the control of the *P21*^*CIP*^ promoter and harboring a *KLF4* site into control and T-lymphoblasts and subsequently incubated for 72 h at 37°C in humidified 5% CO_2_ atmosphere. Transfected control cells displayed increased transcriptional activity of the reporter construct containing the *P21*^*CIP*^ promoter compared with T-lymphoblasts (Figure 4I), suggesting that mutant *KLF4* protein in T-ALL loses its ability to bind to the *KLF4*-binding site present in the *P21*^*CIP*^ promoter, and is thus unable to induce *P21*^*CIP*^. In contrast, wild-type *KLF4* protein bound to its putative site in the *P21*^*CIP*^ promoter and induced its expression in control T-cells.

### Role of *SP1* in *KLF4*-mediated gene expression

**Figure 5:**
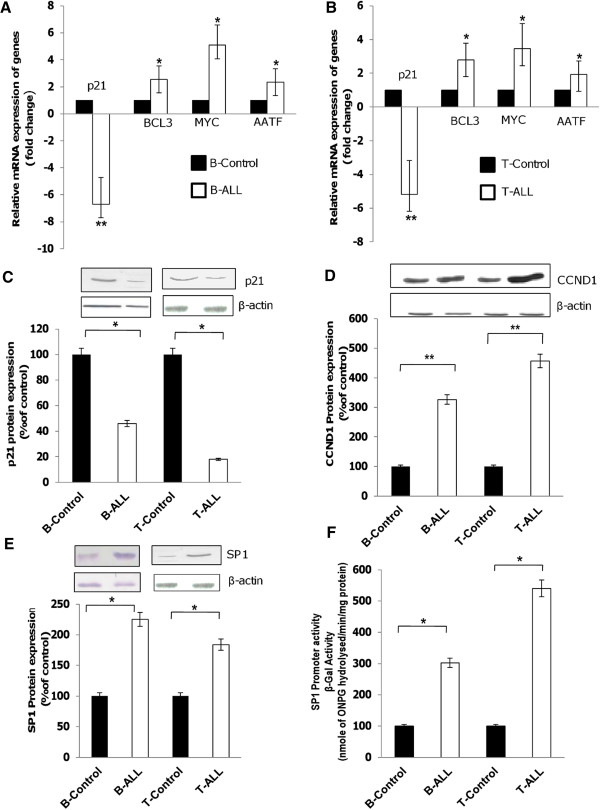
**Expression levels of genes involved in cell cycle suppression (*KLF4 *, *P21***^***CIP***^**), cell proliferation (*MYC, CCND1, BCL3, SP1 *) and apoptosis (*AATF *) in pediatric acute lymphoblastic leukemias. (A-B)** qRT-PCR analysis for expression level of genes including *P21*^*CIP*^, *BCL3, MYC and AATF* in B- and T-cells from patients with pediatric ALL compared with the corresponding controls. Expression was normalized to β-actin and each bar represents mean ± S.D of the experiment performed in triplicate; **P < 0.01; *P < 0.05 relative to control B and T cells. **(C-E)** Western blotting to determine protein expression levels of *P21*^*CIP*^**(C)**, *CCND1***(D)** and *SP1***(E)** in both B- and T-lineage blasts compared with corresponding controls. Expression was normalized to β-actin and protein band intensities were determined using Scion Image Analysis Software. Each bar represents mean ± S.D. of the experiment performed in triplicate; **P < 0.01; *P < 0.05 relative to control B and T cells. **(F)***SP1* transcription factor promoter activity in both B-ALL and T-ALL samples compared with the corresponding controls. B- and T-lymphoblasts were transfected with reporter plasmids containing SP1 response elements, incubated for 72 h at 37°C in humidified 5% CO_2_ atmosphere. The experiments were repeated thrice and results were reported as relative β-gal activity. Each bar represents mean ± S.D of the experiment performed in triplicate *P < 0.05 relative to control.

*KLF4* has been widely recognized to control cell cycle progression through the induction of *P21*^*CIP*^ and subsequent repression of *CCND1* expression [20,21]. It also represses transcription factor *SP1* expression, which regulates genes involved in apoptosis (*AATF*) and cell proliferation (*MYC* and *BCL3*) [22,23]. We therefore investigated the relationship between miR-2909-modulated *KLF4* and the genes involved in cellular proliferation and apoptosis. Both B- and T-lymphoblasts derived from pediatric ALL patients exhibited significantly lower expression levels of *P21*^*CIP*^ mRNA and protein (Figure 5A and C), while mRNA levels of *BCL3*, *MYC* and *AATF* were significantly upregulated (Figure 5A and B). Previous reports suggest it is likely that the low expression levels of *P21*^*CIP*^ were responsible for the overexpression of *CCND1* in both B-ALL and T-ALL (Figure 5D). However, the molecular regulation of *KLF4*-driven genes such as *BCL3, MYC* and *AATF* remains unclear. Given that the promoter regions of all the above-mentioned genes contain *SP1*-binding sites (data not shown) and *KLF4* has the ability to downregulate *SP1* expression, we explored the role of *SP1* in this molecular regulation. *SP1* protein levels were significantly elevated in both B-ALL and T-ALL samples (Figure 5E). To confirm that the elevated *SP1* levels were transcriptionally active, we transfected B- and T-lymphoblasts with reporter plasmids containing *SP1* response elements and found that *SP1* promoter activity was dramatically increased in both B- and T-lineage blasts compared with corresponding controls (Figure 5F). These results indicate that the reduced expression levels of *KLF4* in B-ALL and mutant *KLF4* in T-ALL were unable to repress *SP1* expression, resulting in increased *SP1* levels, which in turn upregulated *BCL3*, *MYC*, and *AATF* expression in both cell types.

### miR-2909 regulates cell cycle and apoptosis in B- but not T-lymphoblasts from patients with pediatric ALL

**Figure 6:**
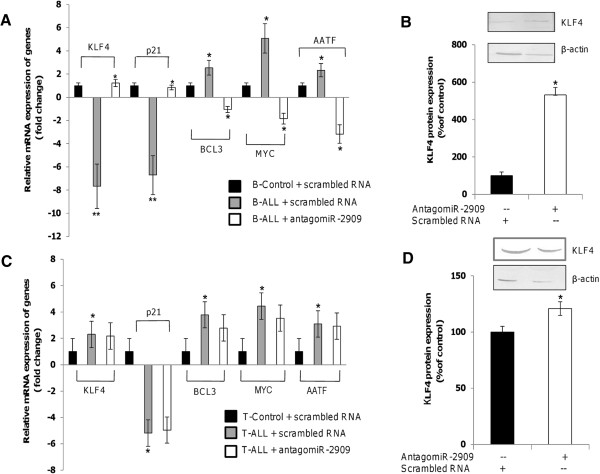
**Effect of miR-2909 knockdown on the expression level of various genes in pediatric patients with B-ALL and T-ALL. (A,C).** qRT-PCR analysis of genes for *KLF4*, *P21*^*CIP*^, *BCL3, MYC, AATF* in B- **(A)** and T-lymphoblasts **(C)** transfected with antagomiR-2909 (50nM) compared with lymphoblasts transfected with control scrambled RNA (50nM). The data representation of mRNA expression levels of the above-mentioned genes in B- and T-lymphoblasts were compared with control B- and T-cells incubated for 48 h at 37°C in humidified 5% CO_2_ atmosphere. **(B,D)***KLF4* protein levels in antagomiR-2909 transfected B- **(B)** and T-lymphoblasts **(D)** compared with lymphoblasts transfected with scrambled RNA. Densitometric analysis of protein bands was carried out using Scion Image Analysis Software. Each bar represents mean ± S.D of the experiment performed in triplicate *P < 0.05 relative to control.

We wanted to determine if the above-mentioned genes were upregulated as a consequence of miR-2909 overexpression in B-ALL. B-lymphoblasts were transfected with antagomiR-2909 to knockdown miR-2909 expression, resulting in significant upregulation of *KLF4* and *P21*^*CIP*^ and downregulation of *MYC*, *BCL3* and *AATF* mRNAs compared with B-lymphoblasts transfected with control scrambled RNA (Figure 6A). *KLF4* protein levels were also significantly increased in these cells (Figure 6B). In contrast, antagomiR-2909-transfection of T-lymphoblasts had no significant effect on any of the above genes or on *KLF4* protein levels compared with T-lymphoblasts transfected with control scrambled RNA (Figure 6C and D). These results thus revealed that miR-2909 plays a key role in the regulation of *P21*^*CIP*^, *KLF4*, *AATF*, *BCL3*, *MYC* and *CCND1*, which control the cell cycle and apoptosis in B-ALL, while miR-2909 loses the capacity to regulate the expression of these genes in T-ALL because they harbor a mutated miR-2909-binding site in the 3′UTR of *KLF4* and a mutated *KLF4* protein.

**Figure 7:**
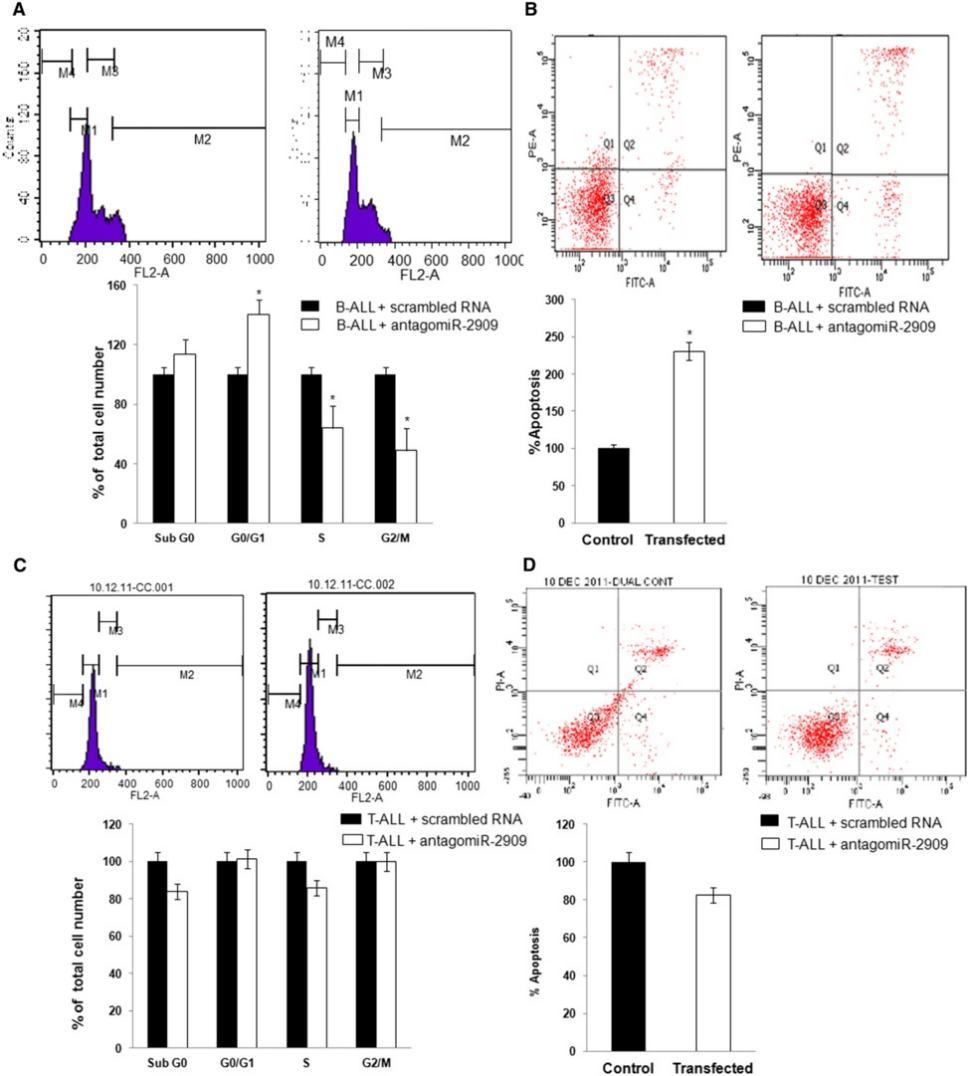
**Effect of miR-2909 knockdown on cell cycle and apoptosis in both B-ALL and T-ALL. (A,C)** Cell cycle analysis of B- **(A)** and T-lympoblasts **(C)** transfected with antagomiR-2909 (50nM) compared with control scrambled RNA-transfected lymphoblasts for 48 h. The percentage of cells in the G1 and S phases of the cell cycle was calculated with Cell Quest Pro Software. **(B,D)** Flow-cytometric detection of apoptosis in B- **(B)** and T-lymphoblasts from patients with pediatric ALL **(D)** relative to control scrambled RNA-transfected lymphoblasts for 48 h. Each bar represents mean ± S.D of the experiment performed in triplicate; *P < 0.05 with respect to control.

We also evaluated the physiological relevance of the miR-2909-regulated genes in the control of the cell cycle and apoptosis in both B-ALL and T-ALL using flow cytometry analysis of antagomiR-2909-transfected cells. The percentage of cells in G1 phase of the cell cycle was dramatically increased, whereas the percentage of cells in S phase was markedly reduced, indicating cell cycle arrest at G1–S phase (Figure 7A) in B-lymphoblasts transfected with antagomiR-2909 compared with control scrambled RNA-transfected B-lymphoblasts. Additionally, antagomiR-2909-transfected B-lymphoblasts showed significantly increased apoptosis as a consequence of downregulation of the *AATF* gene (Figure 7B). In contrast, suppression of miR-2909 expression in T-lymphoblasts by antagomiR-2909 transfection had no effect on the percentage of cells in the G1 and S phases of the cell cycle (Figure 7C) and apoptosis (Figure 7D) relative to T-lymphoblasts transfected with scrambled RNA, further confirming that the mutant *KLF4* in T-ALL was non-functional.

### Overexpression of miR-2909 in HeLa cells induces the expression of oncogenes

**Figure 8:**
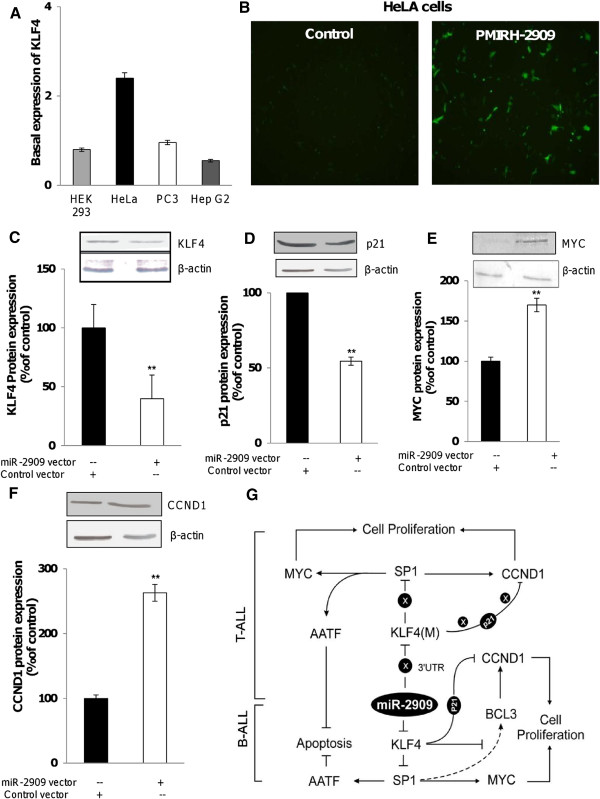
**Overexpression of miR-2909 in HeLa cells confirms its oncogenic properties. (A)** Endogenous levels of *KLF4* protein in indicated cell lines. **(B)** Representative fluorescent microscopic image indicating GFP expression in HeLa cells transfected with pMIRH-2909 expression vector compared with control null vector containing scrambled sequence. **(C-F)** Protein expression levels of *KLF4***(C)**, *P21*^*CIP*^**(D)**, *MYC***(E)** and *CCND1***(F)** in HeLa cells transfected with PMIRH-2909 compared with controls. Densitometric analysis of protein bands was performed using Scion Image Analysis Software. β-actin was used as an invariant control; Each bar represents mean ± S.D. of the experiment performed in triplicate; *P < 0.05 relative to control. **(G)** Schematic model to summarize the critical role of miR-2909 dependent pathway in B-ALL and T-ALL. As shown, miR-2909 is functionally active and regulates *KLF4* and other genes involved in cell cycle progression and apoptosis in pediatric B-ALL while its control over the regulation of *KLF4* and other genes is lost because of mutations in the *KLF4* 3′UTR, which includes the miR-2909 binding site and the altered third zinc finger motif sequence of *KLF4* in pediatric T-ALL.

The elevated levels of miR-2909 in B-ALL reduced expression of the tumor suppressor *KLF4*, thus signifying its oncogenic potential. To investigate its oncogenic nature further, HeLa cells, which express low levels of miR-2909 (Figure 2B) and high levels of *KLF4*, were transfected with the PMIRH-2909 expression vector to increase miR-2909 expression (Figure 8A and B), and protein expression levels of *KLF4*, MYC, *P21*^*CIP*^, and *CCND1* were analyzed. *KLF4* and *P21*^*CIP*^ expression levels were decreased (Figure 8C and D) and MYC and *CCND1* levels were increased (Figure 8E and F) in HeLa cells transfected with PMIRH-2909 compared with controls. These results confirm that miR-2909-mediated suppression of *KLF4* results in failure of *P21*^*CIP*^ induction and promotion of cell proliferation through upregulated expression of *CCND1* and *MYC* proteins.

## Discussion

Mounting evidence has established *KLF4* as a transcriptional activator, repressor, tumor suppressor and an oncogene, depending on its genetic context [8]. However, the exact molecular mechanisms whereby *KLF4* fulfills these roles remain unknown. Moreover, the pathway regulating *KLF4* expression has not been well studied. The present study therefore aimed to investigate the miR-2909-mediated regulation of *KLF4* and its downstream functions, especially cell cycle regulation and apoptosis, in both patients with B- and T-ALL. To the best of our knowledge, the results provide the first evidence for two novel mutations in the *KLF4* gene in T-ALL: a mutation in the 3′UTR resulted in loss of miR-2909-mediated regulation, and mutation in the Zf1/Zf3 motif rendered *KLF4* transcriptionally inactive. In contrast, *KLF4* is regulated post-transcriptionally by miR-2909, and suppression of its expression resulted in loss of *KLF4* tumor suppressor activity in pediatric B-ALL.

Previous studies involving a variety of epithelial tumors have shown that the expression of the zinc-finger transcription factor *KLF4* is silenced by promoter hypermethylation [9]. *KLF4* is also known to be inactivated by methylation in adult T-cell leukemia [24]. Recent studies have revealed direct correlations between altered miRNA levels and progression of hematological malignancies, such as the effects of downregulation of miR-15a and miR-16-1 in chronic lymphocytic leukemia [13] and amplification of miR-155 in B-cell lymphoma, Hodgkin’s lymphoma [25], Burkitt’s lymphoma [26] and in human breast cancer cell lines [27]. However, the role of miRNAs in the pathogeneses of pediatric B- and T-cell ALL remains unknown. Earlier studies from our laboratory reported that the *AATF* gene encodes miR-2909, the functional importance of which remains unexplored in terms of the pathogeneses of B- and T-cell ALLs. We therefore investigated the expression of miR-2909 and its regulation of its target gene *KLF4*. miR-2909 levels were significantly elevated in both B- and T-ALL compared with corresponding age-matched control subjects. Moreover, we found a reciprocal relationship between miR-2909 and *KLF4* expression in B-ALL, but not in T-ALL, which displayed significantly higher levels of *KLF4* expression despite elevated levels of miR-2909. The observed low levels of *KLF4* in B-ALL compared with healthy subjects were in accordance with a previous report [9].

We subsequently explored the possibility that patients with T-ALL may harbor mutations in the *KLF4* 3′UTR, which includes the miR-2909 binding site. Sequence analysis of the 3′UTR revealed altered nucleotides in the ‘seed region’, which is important for target specificity, in T-ALL but not in B-ALL patients. We further demonstrated that miR-2909 targeted the *KLF4* 3′UTR isolated from B-ALL patients, resulting in repression of *KLF4* protein, but not that isolated from T-ALL patients with the mutated miR-2909 binding site, leading to increased levels of *KLF4* protein. Using an miRNASelect pMIR-GFP reporter assay, we confirmed that miR-2909 targeted *KLF4* in B-ALL but not in T-ALL by constructing plasmids containing the 3′UTR and the miR-2909 target site from the B-cell (pGFP-*KLF4*-3′UTR-B) and T-cell lineages (pGFP-*KLF4*-3′UTR-T). Transfection of the pGFP-*KLF4*-3′UTR-B plasmid into HEK 293 cells resulted in a 52.07% reduction in GFP expression, whereas cells expressing pGFP-*KLF4*-3′UTR-T showed no noticeable difference compared with cells transfected with pGFP plasmid without the 3′UTR insert. These results clearly revealed the ability of miR-2909 to repress *KLF4* expression in pediatric ALL B-cell, but not T-cell lineages. Our results thus highlight the molecular differences in miR-2909-mediated differential regulation of *KLF4* between B- and T-ALL. Importantly, our results identifying *KLF4* as a target of miR-2909 in B-ALL are similar to published reports of miRNA-145-mediated repression of *KLF4* in human embryonic stem cells [28] and miR-10b regulation of *KLF4* in human esophageal cancer cell lines [29]. However, these results in pediatric T-ALL provide the first functional demonstration of loss of repression of any target gene as a result of a mutated seed sequence for any miRNA in any disease.

We also explored why elevated *KLF4* levels failed to control excessive proliferation in T-ALL patients by amplifying the full-length *KLF4* sequences from B- and T-ALL samples. Most patients with B-ALL expressed *KLF4* isoform 1, while T-ALL patients expressed isoform 2. However, the precise contributions of each isoform to the overall functions of *KLF4* are not well understood and it will be interesting to validate these findings in a larger cohort to improve our understanding of its implications for the pathogeneses of ALL. In addition to identifying the isoforms present in B- and T-ALL, we also discovered nucleotide insertions or deletions in the Zf1 or Zf3 motif in pediatric T-ALL samples, resulting in frameshift mutations and complete alteration of the zinc-finger motif sequence, which could possibly destroy its DNA-binding affinity.

The crystal structure of the zinc-finger domain of *KLF4* bound to target DNA was recently reported [30], and we exploited this structural information to construct homology models and perform docking studies of wild-type and mutant *KLF4* with the 10-bp *KLF4* target DNA sequence (5′-cgggcggggc-3′). The modeled structure of the Zf3 motif in the mutant *KLF4* revealed the replacement of three key residues (C462V, C465M and H482F) involved in coordination with zinc. Moreover, the docking results demonstrated that mutant *KLF4* was unable to interact with its 10-bp target sequence in the *p21*^*CIP*^ gene promoter, while wild-type *KLF4* displayed cation-π and hydrogen-bonding interactions with its target sequence in controls. These results clearly demonstrate the importance of the Zf3 motif in *KLF4*-mediated functional regulation of its target gene. These findings are consistent with a previous study demonstrating that *KLF4*-mediated macrophage differentiation was primarily controlled by the two C-terminal Zf2 and Zf3 motifs, while Zf1 imparted little specificity [30]. More importantly, we provided experimental proof to support the docking studies, showing that mutant *KLF4* in T-lymphoblasts was unable to induce *p21*^*CIP*^ promoter activity, while *KLF4* in control T-cells increased *p21*^*CIP*^ promoter reporter activity. A similar phenomenon was reported in colorectal cancer cell lines, where *KLF4* mutations resulted in reduced *p21*^*CIP*^ promoter activity [31].

*KLF4* acts as cell cycle regulator and functions as a tumor suppressor through its ability to induce p21^CIP^[20] and suppress *SP1* expression [22]. Based on these findings, we studied the expression levels of genes involved in this process in ALL patients. We observed significant reductions in *p21*^*CIP*^ and increases in *SP1* in B-ALL as a result of suppressed *KLF4* expression. In contrast, elevated levels of mutant *KLF4* failed to induce *p21*^*CIP*^ or inhibit *SP1* expression in T-ALL, leading to low levels of *p21*^*CIP*^ and high levels of *SP1*, respectively. Moreover, the increased levels of *SP1* were associated with increased transcriptional activity of a reporter construct containing an *SP1* response element in both B-and T-ALL subtypes. Earlier work from our laboratory showed that *SP1* upregulated *AATF* and *MYC* gene in Jurkat cells [23]. Additionally, bioinformatics analysis revealed the presence of an *SP1* response element in the promoter regions of *AATF*, *MYC* and *BCL3* (data not shown). Consistent with these results, we also confirmed that upregulated *SP1* levels resulted in increased *BCL3*, *AATF* and *MYC* expression in both B- and T-ALL subtypes. Our results therefore suggest that *KLF4*-mediated regulation of these genes most likely occurs through *SP1* in both B- and T-ALL. The current study also assessed the key role of miR-2909 in the regulation of the above-mentioned genes. Results in B-lymphoblasts transfected with antagomiR-2909 confirmed that miR-2909 regulates these genes through *KLF4*. Transfection of pediatric B-lymphoblasts with antagomiR-2909 for 48 h resulted in significant upregulation of *KLF4* expression. Furthermore, antagomiR-2909-transfected B-lymphoblasts revealed significant repression of the *AATF*, *MYC* and *BCL3* genes. In contrast, antagomiR-2909-transfection had no such effects in pediatric T-lymphoblasts, as a result of failure of miR-2909 to bind to the mutated 3′UTR of *KLF4* and mutated *KLF4* protein. We subsequently tested the functional relevance of increased *KLF4* in antagomiR-2909-transfected B- and T-lymphoblasts. B-lymphoblasts showed cell cycle arrest at G1/S phase, most likely through increased expression of *p21*^*CIP*^, and increased apoptosis, possibly resulting from *AATF* suppression. However, no such changes were seen in antagomiR-2909-transfected pediatric T-lymphoblasts, despite increased levels of *KLF4*, which was rendered transcriptionally inactive as a result of the altered Zf3 motif, which lacked DNA-binding activity. In addition, antagomiR-2909-transfected B-lymphoblasts also exhibited decreased mRNA expression of *MYC* and *BCL3* mRNA expression levels, which may reduce cell proliferation. These results were similar to other reported studies showing increased expression of *p21*^*CIP*^ and decreased expression of *MYC* and *cyclin D2* in pro/pre-B cells expressing *KLF4*[32]. Furthermore, miR-2909 overexpression and its ability to target the tumor suppressor *KLF4* suggest that it displays oncogenic properties in B-ALL. To prove this, we overexpressed miR-2909 in HeLa cells to silence *KLF4* expression. This resulted in significantly increased *CCND1* and *MYC* and decreased p21^CIP^ protein levels. These results are consistent with previous findings, which showed that *KLF4-*silencing in HeLa cells promoted cell growth and tumor formation [33].

Collectively, our experimental results suggest the existence of a molecular pathway as depicted in Figure 8G, which summarizes the differential nature of the miR-2909-*KLF4* axis in B-ALL and T-ALL. The proposed pathway explains how high mutant *KLF4* protein expression in T-ALL subjects is unable to induce *p21*^*CIP*^, which plays a major role in inhibiting *CCND1* expression. Mutant *KLF4* protein also failed to repress *SP1* expression, a gene known to be downregulated by *KLF4*, in parallel with upregulation of the oncogenes *MYC*, *BCL3* and the anti-apoptotic *AATF*, potentially leading to unbridled T-cell transformation. A similar phenomenon is proposed in B-ALL subjects, wherein downregulation of *KLF4* protein by miR-2909 overexpression results in B-cell transformation. Significantly, our studies revealed two breakpoints in the miR-2909-*KLF4* axis caused by mutation in the 3′UTR-*KLF4* and the altered Zf3 motif sequence, leading to loss of miR-2909 binding to the *KLF4* 3′UTR and loss of *KLF4* binding to DNA sequences in target genes in T-cell ALL subjects. Moreover, structural modeling of the zinc-finger motif of *KLF4* uncovered the precise molecular basis of loss of DNA binding in T-ALL, associated with alterations of three key amino acid residues involved in zinc coordination. Overall, our findings suggest that *KLF4* function is compromised in T-cells derived from patients with pediatric T-ALL because of alteration of the Zf3 motif sequence as a result of a frameshift mutation. Furthermore, its regulation by miR-2909 is impaired because of a mutation in the miR-2909 target sequence present in the 3′UTR of the *KLF4* mRNA. In B-ALL subjects, *KLF4* function is compromised because of miR-2909-dependent downregulation. *KLF4* is therefore unable to act as a tumor suppressor gene in either pediatric B-ALL or T-ALL. These results raise the possibility of the existence of similar *KLF4* mutations in other tissues. However it was not possible to investigate the presence of such mutations in other tissues from the study patients because of ethical considerations.

## Conclusions

The present study discovered that elevated levels of the novel miR-2909 target the tumor suppressor *KLF4*, which regulates the cell cycle and apoptosis in pediatric ALL. miR-2909-mediated downregulation resulted in loss of *KLF4* activity in B-ALL. In contrast, miR-2909 failed to regulate *KLF4* expression in T-ALL because of mutations in the *KLF4* 3′UTR, which includes the miR-2909 binding site. The molecular defect responsible for loss of *KLF4* function in T-ALL despite its elevated level, lies within the Zf3 motif, which is altered as a result of a frameshift mutation. Homology modeling/docking studies and *p21*^*CIP*^ promoter activity confirmed the lack of functional activity of mutant *KLF4*. Comprehensive sequence analysis of *KLF4* identified the predominance of isoform 1 in most patients with pediatric B-ALL, and of isoform 2 in patients with T-ALL. These results demonstrate the existence of a novel miR-2909-*KLF4* molecular axis able to differentiate between B- and T-ALL pathogeneses, and which may provide a new diagnostic/prognostic marker to evaluate the pathogenesis of ALL in pediatric subjects. Our results also suggest that screening for these two mutations could be developed as a diagnostic strategy to differentiate between these two ALL subtypes. Overall, this study provides the basis for further research that could lead to a novel therapeutic approach for patients with B-and T-cell ALL.

## Materials and methods

### Materials

The reagents were procured as follows: MiniMACS™ Starting Kit (Miltenyi Biotech, Auburn, CA); miRNeasy mini kit, miScript Reverse transcription kit, miScript SYBR Green kit, Universal primer for amplifying miR-2909, Pfu polymerase (Fermentas, Vilnius, Lithuania); Qiaquick PCR purification kit (Qiagen, Valencia, CA); pBlue TOPO reporter vector, Lipofectamine 2000 and β Gal Assay Kit (Invitrogen,Carlsbad, USA); anti-KLF4 (Abcam), anti-p21^CIP^, anti-SP1, anti-MYC, anti-CCND1, anti-β actin; Annexin V-FITC apoptosis kit (Sigma-Aldrich, St.Louis, MO, USA); pMIR-GFP Reporter Vector (Cell Biolabs, San Diego, CA, USA); miRCURY LNA™ miR-2909 power inhibitor (EXIQON, Denmark); PMIRH-2909 lentiviral construct (System Biosciences, CA, USA).

### Patient samples

The pediatric B-ALL, T-ALL samples and age-matched control samples were obtained from Advanced Pediatric Centre, Post Graduate Institute of Medical Education and Research (PGIMER), Chandigarh, India with prior consent from their guardian through ethical approval by the Institutional Review Board of PGIMER. Total samples of B-ALL and T-ALL were 30 and 20 respectively. Age-matched subjects (n = 50) with no manifestations of any haematological malignancy were treated as control. All patients were <14 years of age and flow cytometric immunophenotyping showed mostly cells in the blast/progenitor region (SSC low to moderate, CD45 negative to dim) comprising ~80% of all singlet events acquired. Patients undergoing chemotherapy and cases with mixed phenotype acute leukemia (MPAL) and relapsed cases from pediatric acute lymphoblastic leukaemia (ALL) were excluded in the present study. Diagnosis of pediatric ALL samples into B- and T-cell lineage was evaluated by immunophenotyping using CD19, CD22, CD79a, CD10, CD20, and CD24 for B-ALL and CD7,CD2,CD3,CD5 for T-ALL. Mononuclear cells were isolated using Ficoll-Hypaque density gradient method [34]. B- and T-cells were purified using MiniMACS™ Separator Kit.

### RNA Extraction, cDNA synthesis and qRT-PCR

Total RNA including the small RNA was extracted from patient samples using miRNeasy mini kit in accordance with the manufacturer’s instructions. The quality and quantity of extracted RNAs were analyzed using electrophoresis and optical density measurement at 260 nm; cDNA synthesis was performed via miScript Reverse transcription kit as per suppliers’s instructions. For assaying gene expression, miScript SYBR Green Mix and the Real-time PCR (Stratagene, San Diego, CA, USA) were used. The qRT-PCR reaction was performed with a starting temperature of 95°C for 10 min, followed by 35 cycles of 45 s at 94°C, 30 s at 56°C, and 45 s at 72°C. The small non-coding nuclear RNA U6 and β-actin were used as an invariant controls for normalizing the expression of miR-2909 and other genes respectively. The 2-^ΔΔCT^ method was used to calculate the relative expression of target genes.

### Immunoblotting

Total cellular protein was extracted using Laemmli’s buffer [35] and the protein levels of *KLF4, p21*^*CIP*^*, SP1, MYC and CCND1* was determined through western blotting using appropriate antibodies as described previously [36]. β-actin antibody was used as an internal control. Scion Image Analysis software was used for densitometry analysis and the results were expressed as intensity ratio of target protein to β-actin protein taken as arbitrary unit.

### DNA sequencing

Primer sets were designed to amplify the full coding region and 3′untranslated region of *KLF4* in ALL samples using *Pfu* polymerase. The resultant PCR products were purified using Qiaquick PCR purification kit and sequenced to detect the presence of any genetic aberration(s) in KLF4 in samples from pediatric patients with ALL. The sequence data was analysed using Cluster X 2.0.12 Software (http://www.clustal.org/clustal2) [37].

### Plasmid constructs and reporter assays

Full length 3′UTR of KLF4 in B-ALL was cloned into miRNASelect™ pMIR GFP reporter vector; designated as pGFP-KLF4-3′UTR-B which carried no substitution of nucleotides within miR-2909 target site in KLF4 3′UTR. Mutant 3′UTR of KLF4 present in T-ALL was named as pGFP-KLF4-3′UTR-T with substitution of nucleotides within core binding site in KLF4 3′UTR. The plasmid constructs were transfected in HEK-293 cells. After 48 h, fluoresence microscopy and FACS analysis was performed to quantitate the number of cells expressing GFP. For *p21*^*CIP*^ promoter analysis, promoter sequence of *p21*^*CIP*^ with putative *KLF4* binding site was cloned into pBlue TOPO reporter vector with subsequent transfection of β-gal construct into control and T- lymphoblasts. For analysis of SP1 transcriptional activity, B- and T-lymphoblasts with reporter plasmids containing *SP1* response elements were transfected. β galactosidase activity was measured 72 h after transfection. To knockdown miR-2909 expression, leukemia cells were transfected with miRCURY LNA™ miR-2909 power inhibitor. To increase miR-2909 expression, HeLa cells were transfected with the PMIRH-2909 expression vector. All the transfections were performed with Lipofectamine 2000 transfection reagent according to the manufacturer’s instructions.

### Cell cycle analysis and apoptosis assays by Flow cytometry

Cell cycle analysis and apoptotic assays was done on leukemia cells transfected with antagomiR-2909 (50 nM) and scrambled RNA (50 nM) for 48 h in RPMI 1640 medium supplemented with 10% FBS, 100 U/ml penicillin and 100 μg/ml streptomycin under 5% CO_2_ at 37°C. For cell cycle experiments, cells were fixed in 70% ethanol and stained with PI. Cells percentage at different phases were analysed with FACSCalibur cytometer and Cell Quest Pro software (Becton Dickinson, NJ, USA). For apoptosis assays, cells were stained with FITC Annexin V coupled with propidium iodide and apoptosis was measured using BD FACS Diva Software (Becton Dickinson, FACS Canto II).

### KLF4 structural model & docking with target DNA

The structural models of zinc finger motifs of wild-type and mutant KLF4 were modeled with template PDB ID: 2WBUA. The Homology models were built using MODELLER (9.9) [38]. Model validation was performed using Verify-3D (http://nihserver.mbi.ucla.edu/Verify_3D/) [39] and PROCHECK [40]. The quality of the final models was evaluated from Ramachandran plot (Additional file 3: Figure S3B). Molecular visualization and structural alignment was done using CHIMERA http://bioinformatics.org/wiki/Chimera[41] and PYMOL http://www.pymol.org/[42]. Target DNA sequence (5′-cgggcggggc-3′) in *p21*^*CIP*^ promoter was modeled into B-FORM using DNA analysis servers [43]. Docking studies were performed using High Ambiguity-Driven bimolecular Docking (HADDOCK) under solvated conditions [44,45]. Cation-π interactions were analysed with CAPTURE http://capture.caltech.edu/[46].

### Statistical analysis

Statistical analyses were performed by SPSS Windows version 19. Data was expressed as mean ± S.D of the experiments performed in triplicate. Student’s *t* test or Mann-Whitney-Wilcoxon test was performed to determine the significance of difference between two groups. Differences were considered significant at p < 0.01 and p < 0.05.

## Abbreviations

ALL: Acute lymphoblastic leukemia; miR: microRNA; B-ALL: B-cell lineage acute lymphoblastic leukemia; T-ALL: T-cell lineage acute lymphoblastic leukemia; AATF: Anti-apoptotic transcription factor; KLF4: Kruppel like factor transcription factor 4; BCL3: B-cell lymphoma 3-encoded protein; CCND1: Cyclin D1; Zf1: First zinc finger motif; Zf2: Second zinc finger motif; Zf3: Third zinc finger motif; *SP1*: Specificity protein transcription factor-1; UTR: Untranslated region; CD: Cluster of differentiation; GFP: Green fluorescent protein; β-gal: β-galactosidase.

## Competing interests

The authors have no competing interests.

## Authors’ contributions

The work was conceived by DK and executed by DM and NC. Patients were supplied by RK. All authors read and approved the final manuscript.

## Supplementary Material

Additional file 1: Figure S1 Sequence analysis of *KLF4* coding region in pediatric T-ALL samples. **(A-F)** Representative DNA sequence alignment of *KLF4* coding region (corresponding to the three zinc finger motifs in exon 5) in T-ALL samples. NCBI sequence is shown for comparison. Sequence analyses indicate insertion **(A)**, deletion **(B-F)** of nucleotides in the first or third zinc finger motif (Zf1, Zf3) of *KLF4* in T-ALL samples. These genetic aberration(s) changed the entire reading frame, altering the sequence of *KLF4* third zinc finger motif and potentially destroying its DNA-binding affinity **(G)** Protein sequence alignment of these same 6 T-ALL samples with respect to NCBI. Identical Zf1 and Zf2 motif in pediatric T-ALL samples is highlighted. Click here for file

Additional file 2: Figure S2 Sequence analysis of *KLF4* coding region in pediatric B-ALL samples. **(A,B)** Representative DNA **(A)** and protein sequence alignment **(B)** of *KLF4* coding region (corresponding to the three zinc finger motifs; Zf1, Zf2, Zf3 in exon 5) in all B-ALL samples in the present study. NCBI sequence is shown for comparison. Sequence analysis revealed no genetic aberrations in any of the three zinc-fingers regions of KLF4 in samples from pediatric patients with B-ALL, suggesting that the conformation of *KLF4* was unaffected in these patients (sample size 10). Click here for file

Additional file 3: Figure S3 Structural models and docking studies of wild-type and mutant *KLF4***(A)** Predicted residues in the active site of *KLF4* were Arg449, Arg467, Lys453, Gly456, His457, Arg458, Ser470, Arg471 and His474 as given by CASTP server. Most of the active residues reside in the second and third zinc finger motifs; Zf2 and Zf3 as indicated by green dots. **(B)** Ramachandran plot of wild-type and mutant *KLF4* which was built using PROCHECK; wild- type *KLF4* has 82.7% of residues in favoured region and the remaining 16.0% in additionally allowed regions; mutant *KLF4* has 81.4% residues in the most favoured region and remaining 17.1% in additionally allowed regions. **(C-D)** Representative table exhibited the formation of hydrogen bonds between Ser 470 and guanine (at position 11) in wild-type **(C)** and between Arg458 and guanine (at position 3) in mutant **(D)***KLF4***(E-F)** Representative table showed non-covalent interactions, primarily electrostatic hydrophobic and Van der waals forces between protein residues and bases (guanine and cytidine) in wild-type **(E)** and mutant *KLF4***(F)**. DNA bases within the parentheses interact simultaneously with its corresponding protein residue. All the active site residues in wild-type *KLF4* displayed hydrophobic and Van der waal interactions, in contrast only few active site residues in mutant *KLF4* were involved in these interactions. Click here for file
