# Supplementary material for: miR-2909-mediated regulation of KLF4: a novel molecular mechanism for differentiating between B-cell and T-cell pediatric acute lymphoblastic leukemias
Source: Mol Cancer. 2014 Jul 18;13:175. doi: 10.1186/1476-4598-13-175 (PMC4112645; doi:10.1186/1476-4598-13-175)
Supplement: Additional file 1: Figure S1 — Sequence analysis of KLF4 coding region in pediatric T-ALL samples. (A-F) Representative DNA sequence alignment of KLF4 coding region (corresponding to the three zinc finger motifs in exon 5) in T-ALL samples. NCBI sequence is shown for comparison. Sequence analyses indicate insertion (A), deletion (B-F) of nucleotides in the first or third zinc finger motif (Zf1, Zf3) of KLF4 in T-ALL samples. These genetic aberration(s) changed the entire reading frame, altering the sequence of KLF4 third zinc finger motif and potentially destroying its DNA-binding affinity (G) Protein sequence alignment of these same 6 T-ALL samples with respect to NCBI. Identical Zf1 and Zf2 motif in pediatric T-ALL samples is highlighted. [file 1476-4598-13-175-S1.pdf]

miR-2909-mediated regulation of KLF4: a novel molecular mechanism for differentiating between B-cell and T-cell pediatric acute lymphoblastic leukemias

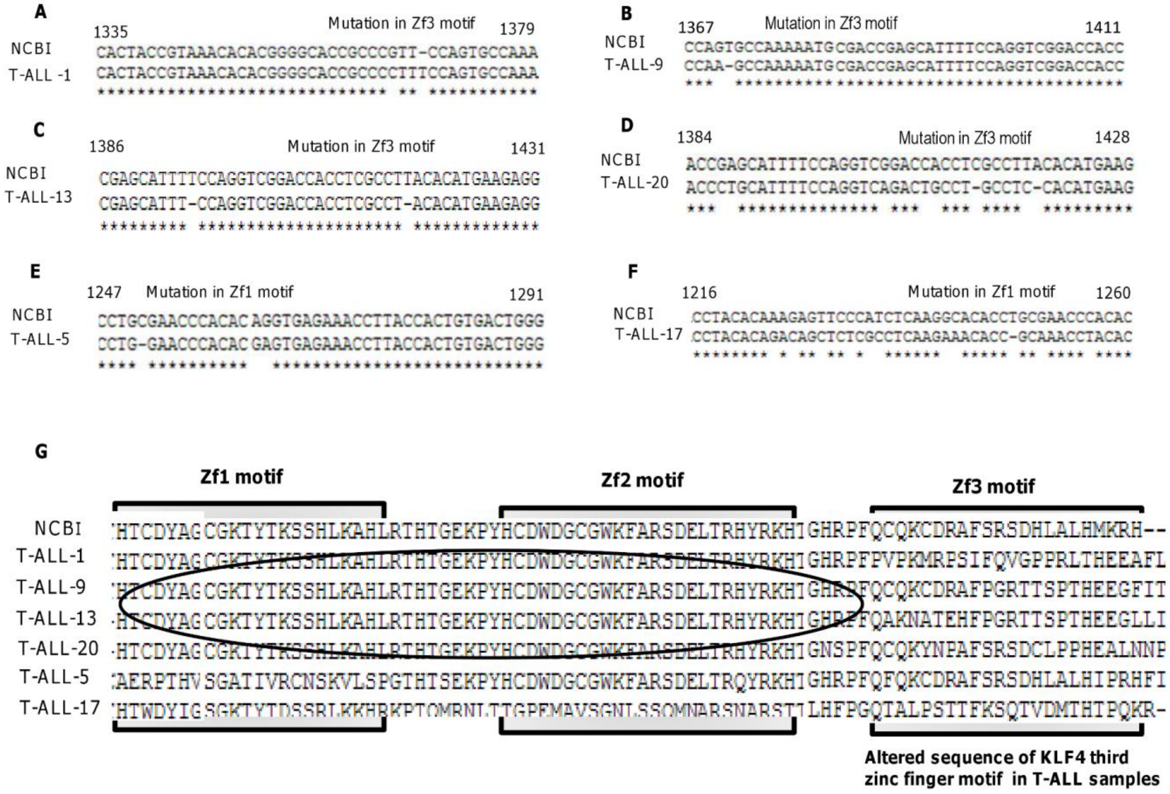

Figure S1. Sequence analysis of *KLF4* coding region in pediatric T-ALL samples. (A-F) Representative DNA sequence alignment of *KLF4* coding region (corresponding to the three zinc finger motifs in exon 5) in T-ALL samples. NCBI sequence is shown for comparison. Sequence analyses indicate insertion **(A)**, deletion **(B-F)** of nucleotides in the first or third zinc finger motif (Zf1, Zf3) of *KLF4* in T-ALL samples. These genetic aberration(s) changed the entire reading frame, altering the sequence of *KLF4* third zinc finger motif and potentially destroying its DNA-binding affinity **(G)** Protein sequence

alignment of these same 6 T-ALL samples with respect to NCBI. Identical Zf1 and Zf2 motif in pediatric T-ALL samples is highlighted.
