# Supplementary material for: miR-2909-mediated regulation of KLF4: a novel molecular mechanism for differentiating between B-cell and T-cell pediatric acute lymphoblastic leukemias
Source: Mol Cancer. 2014 Jul 18;13:175. doi: 10.1186/1476-4598-13-175 (PMC4112645; doi:10.1186/1476-4598-13-175)
Supplement: Additional file 2: Figure S2 — Sequence analysis of KLF4 coding region in pediatric B-ALL samples. (A,B) Representative DNA (A) and protein sequence alignment (B) of KLF4 coding region (corresponding to the three zinc finger motifs; Zf1, Zf2, Zf3 in exon 5) in all B-ALL samples in the present study. NCBI sequence is shown for comparison. Sequence analysis revealed no genetic aberrations in any of the three zinc-fingers regions of KLF4 in samples from pediatric patients with B-ALL, suggesting that the conformation of KLF4 was unaffected in these patients (sample size 10). [file 1476-4598-13-175-S2.pdf]

miR-2909-mediated regulation of KLF4: a novel molecular mechanism for differentiating between B-cell and T-cell pediatric acute lymphoblastic leukemias

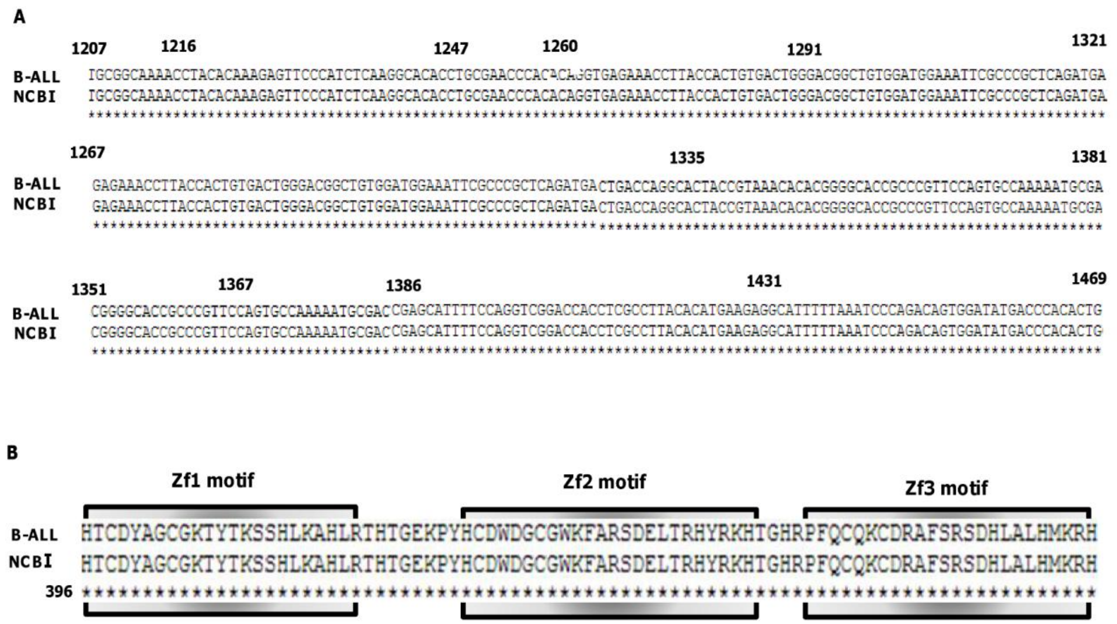

Figure S2. Sequence analysis of *KLF4* coding region in pediatric B-ALL samples. (A,B) Representative DNA **(A)** and protein sequence alignment **(B)** of *KLF4* coding region (corresponding to the three zinc finger motifs; Zf1, Zf2, Zf3 in exon 5) in all B-ALL samples in the present study. NCBI sequence is shown for comparison. Sequence analysis revealed no genetic aberrations in any of the three zinc-fingers regions of KLF4 in samples from pediatric patients with B-ALL, suggesting that the conformation of *KLF4* was unaffected in these patients (sample size 10).
