# Supplementary material for: miR-2909-mediated regulation of KLF4: a novel molecular mechanism for differentiating between B-cell and T-cell pediatric acute lymphoblastic leukemias
Source: Mol Cancer. 2014 Jul 18;13:175. doi: 10.1186/1476-4598-13-175 (PMC4112645; doi:10.1186/1476-4598-13-175)
Supplement: Additional file 3: Figure S3 — Structural models and docking studies of wild-type and mutant KLF4(A) Predicted residues in the active site of KLF4 were Arg449, Arg467, Lys453, Gly456, His457, Arg458, Ser470, Arg471 and His474 as given by CASTP server. Most of the active residues reside in the second and third zinc finger motifs; Zf2 and Zf3 as indicated by green dots. (B) Ramachandran plot of wild-type and mutant KLF4 which was built using PROCHECK; wild- type KLF4 has 82.7% of residues in favoured region and the remaining 16.0% in additionally allowed regions; mutant KLF4 has 81.4% residues in the most favoured region and remaining 17.1% in additionally allowed regions. (C-D) Representative table exhibited the formation of hydrogen bonds between Ser 470 and guanine (at position 11) in wild-type (C) and between Arg458 and guanine (at position 3) in mutant (D)KLF4(E-F) Representative table showed non-covalent interactions, primarily electrostatic hydrophobic and Van der waals forces between protein residues and bases (guanine and cytidine) in wild-type (E) and mutant KLF4(F). DNA bases within the parentheses interact simultaneously with its corresponding protein residue. All the active site residues in wild-type KLF4 displayed hydrophobic and Van der waal interactions, in contrast only few active site residues in mutant KLF4 were involved in these interactions. [file 1476-4598-13-175-S3.pdf]

miR-2909-mediated regulation of KLF4: a novel molecular mechanism for differentiating between B-cell and T-cell pediatric acute lymphoblastic leukemias

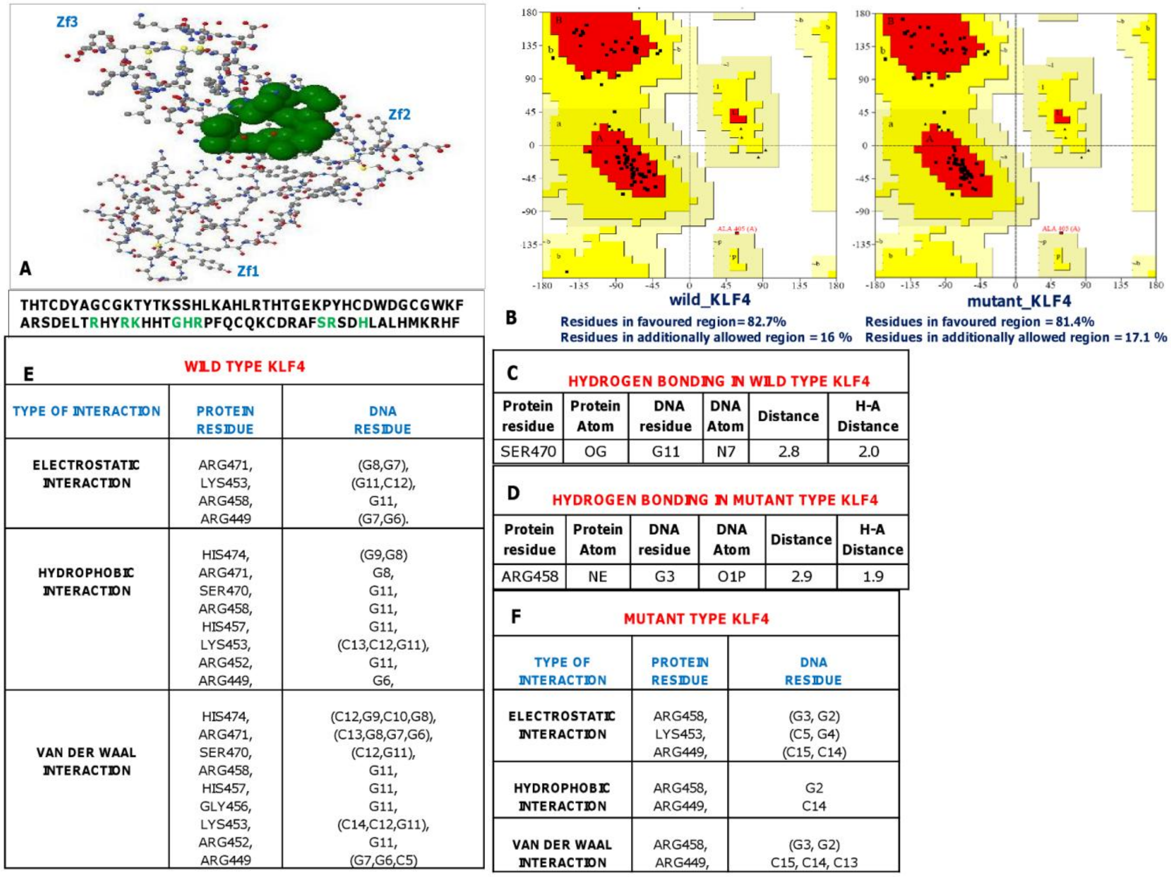

E WILD TYPE KLF4

TYPE OF INTERACTION	PROTEIN RESIDUE	DNA RESIDUE
ELECTROSTATIC INTERACTION	ARG471, LYS453, ARG458, ARG449	(G8,G7), (G11,C12), G11, (G7,G6).
HYDROPHOBIC INTERACTION	HIS474, ARG471, SER470, ARG458, HIS457, LYS453, ARG452, ARG449,	(G9,G8) G8, G11, G11, G11, (C13,C12,G11), G11, G6,
VAN DER WAAL INTERACTION	HIS474, ARG471, SER470, ARG458, HIS457, GLY456, LYS453, ARG452, ARG449	(C12,G9,C10,G8), (C13,G8,G7,G6), (C12,G11), G11, G11, G11, (C14,C12,G11), G11, (G7,G6,C5)

C HYDROGEN BONDING IN WILD TYPE KLF4

Protein residue	Protein Atom	DNA residue	DNA Atom	Distance	H-A Distance
SER470	OG	G11	N7	2.8	2.0

D HYDROGEN BONDING IN MUTANT TYPE KLF4

Protein residue	Protein Atom	DNA residue	DNA Atom	Distance	H-A Distance
ARG458	NE	G3	O1P	2.9	1.9

F MUTANT TYPE KLF4

TYPE OF INTERACTION	PROTEIN RESIDUE	DNA RESIDUE
ELECTROSTATIC INTERACTION	ARG458, LYS453, ARG449,	(G3, G2) (C5, G4) (C15, C14)
HYDROPHOBIC INTERACTION	ARG458, ARG449,	G2 C14
VAN DER WAAL INTERACTION	ARG458, ARG449,	(G3, G2) C15, C14, C13

Figure S3. Structural models and docking studies of wild-type and mutant *KLF4* (A) Predicted residues in the active site of *KLF4* were Arg449, Arg467, Lys453, Gly456, His457, Arg458, Ser470, Arg471 and His474 as given by CASTP server. Most of the active residues reside in the second and third zinc finger motifs; Zf2 and Zf3 as indicated by green dots. **(B)** Ramachandran plot of wild-type and mutant *KLF4* which was built using PROCHECK; wild-type *KLF4* has 82.7 % of residues in favoured region and the remaining 16.0% in additionally allowed regions; mutant *KLF4* has 81.4% residues in the most favoured region

and remaining 17.1% in additionally allowed regions. **(C-D)** Representative table exhibited the formation of hydrogen bonds between Ser 470 and guanine (at position 11) in wild-type **(C)** and between Arg458 and guanine (at position 3) in mutant **(D)** *KLF4* **(E-F)** Representative table showed non-covalent interactions, primarily electrostatic hydrophobic and Van der waals forces between protein residues and bases (guanine and cytidine) in wild-type **(E)** and mutant *KLF4* **(F)**. DNA bases within the parentheses interact simultaneously with its corresponding protein residue. All the active site residues in wild-type *KLF4* displayed hydrophobic and Van der waal interactions, in contrast only few active site residues in mutant *KLF4* were involved in these interactions.
